# Les brûlures oculaires : aspects épidémiologiques, cliniques, thérapeutiques et évolutifs au Centre hospitalier universitaire de Cocody, Côte d'Ivoire

**DOI:** 10.48327/mtsi.v4i1.2024.486

**Published:** 2024-02-12

**Authors:** Chiatse Ellalie KO MAN, Sienou Marguerite Pascaline KONAN MANMI, Reine Prisca AGBOHOUN, Colette KOUASSI-REBOURS, Yves Thierry Constant SOWAGNON, Hermine Cynthia N'DA, Cédric Romarie KOUADIO KOUAO, Laeticia Coralie N'GUESSAN, François Xavier KOUASSI

**Affiliations:** 1 Service dophtalmologie, Centre hospitalier universitaire de Cocody, Département de chirurgie et spécialités chirurgicales, Université Félix Houphouët-Boigny, Abidjan, Côte d'Ivoire; 2 Service dophtalmologie, Centre hospitalier universitaire de Yopougon, Département de chirurgie et spécialités chirurgicales, Université Félix Houphouët-Boigny, Abidjan, Côte d'Ivoire

**Keywords:** Brûlure oculaire, Traumatisme oculaire, Cécité, Cocody, Abidjan, Côte d'Ivoire, Afrique subsaharienne, Ocular burn, Ocular trauma, Blindness, Cocody, Abidjan, Côte d'Ivoire, Sub-Saharan Africa

## Abstract

**Justification:**

Cette étude décrit les caractéristiques socio-démographiques, les aspects cliniques, thérapeutiques et évolutifs des brûlures oculaires afin de contribuer à l'amélioration de leur prise en charge.

**Méthode:**

Étude rétrospective réalisée dans le service d'ophtalmologie du Centre hospitalier universitaire (CHU) de Cocody à Abidjan (Côte d'Ivoire) du 1er janvier 2020 au 31 janvier 2021. Elle a porté sur 49 dossiers de patients victimes de traumatismes oculaires avec 12 cas bilatéraux, soit un total de 61 yeux. Pour chaque patient, les données socio-démographiques, la nature de l'agent traumatisant, les étiologies des brûlures, le stade de brûlure oculaire, l'acuité visuelle de loin sans correction initiale puis finale de l’œil atteint et le traitement ont été recueillis.

**Résultats:**

La part des brûlures oculaires était de 11 % pour 436 cas de traumatismes oculaires ayant consulté dans le service. L’âge moyen des patients était de 27,9 ans ± 14,2 avec des extrêmes de 3 et 60 ans et une prédominance masculine (70 %). Les élèves et étudiants étaient la catégorie socio-professionnelle la plus fréquente (39 %). Les circonstances de survenue étaient dominées par les accidents de travail dans un tiers des cas. Le principal agent traumatisant était chimique, dans 54 % des cas. Le délai moyen de consultation était de 3,5 jours ± 7,9 avec des extrêmes de 1 et 60 jours. Le stade 1 de la classification de Roper-Hall était le stade le plus observé (51 % des cas). L'acuité visuelle initiale de l’œil atteint était inférieure à 1/20 dans 28 % des cas. Le traitement était essentiellement médical. Un tiers des yeux traités avaient une acuité finale inférieure à 1/20.

**Conclusion:**

Le pronostic visuel est conditionné par les stades de brûlure, les étiologies et le délai de consultation, variables selon les origines sociales et géographiques.

## Abstract (en)

**Justification:**

This study describes the socio-demographic characteristics, clinical, therapeutic, and evolutionary aspects of ocular burns to contribute to the improvement of their management.

**Method:**

A retrospective study was conducted in the Ophthalmology Department of the Cocody University Hospital (CHU) in Abidjan, Côte d'Ivoire, from January 1, 2020, to January 31, 2021. It focused on 49 patient records with ocular trauma, including 12 bilateral cases, totaling 61 eyes. For each patient, socio-demographic data, the nature of the traumatic agent, burn etiologies, ocular burn stage, initial and final uncorrected visual acuity of the affected eye, and treatment were collected.

**Results:**

The proportion of ocular burns was 11% out of 436 cases of ocular trauma that consulted in the department. The average age of patients was 27.9 years ± 14.2, ranging from 3 to 60 years, with a male predominance (70%). Students were the most frequent socio-professional category (39%). Work-related accidents were the predominant circumstances, in a third of cases. Chemical agents were the main traumatic agents, in 54% of cases. The average consultation time was 3.5 days ± 7.9, ranging from 1 to 60 days. Stage 1 of the Roper-Hall classification was the most observed stage (51% of cases). Initial visual acuity of the affected eye was less than 1/20 in 28% of cases. Treatment was mainly medical, and a third of treated eyes had a final acuity less than 1/20.

**Conclusion:**

Visual prognosis is influenced by burn stages, etiologies, and consultation time, varying according to social and geographical origins.

## Introduction

Les brûlures oculaires sont une urgence ophtalmologique. Elles peuvent être chimiques ou thermiques et représentent 7 à 18 % des traumatismes oculaires [5]. Il s'agit le plus souvent d'accidents de travail, domestiques, de loisir et d'agressions [3]. Les brûlures chimiques sont de loin les plus fréquentes [5]. Le pronostic visuel est modulé par la nature de l'agent responsable, la durée de contact de l'agent causal et la rapidité de la prise en charge initiale.

Dans notre pratique quotidienne, une augmentation de la fréquence des brûlures oculaires a été observée. Cela nous a amenés à décrire les aspects épidémiologiques, cliniques, thérapeutiques et pronostiques des patients présentant une brûlure oculaire au CHU de Cocody en vue d'améliorer la prise en charge.

## Méthode

Cette étude rétrospective à visée descriptive et analytique s'est déroulée dans le service d'ophtalmologie du Centre hospitalier universitaire (CHU) de Cocody à Abidjan (Côte d'Ivoire) sur une période allant du 1^er^ janvier 2020 au 31 janvier 2021, soit une durée de 13 mois. Elle a porté sur 49 dossiers de patients victimes de brûlures oculaires avec 12 cas bilatéraux, soit un total de 61 yeux.

Tous les patients pris en charge pour brûlure oculaire avérée pendant notre période d’étude ont été inclus. Les données ont été extraites des dossiers médicaux à partir d'un questionnaire portant sur :

- Les données socio-démographiques (âge, sexe, activités, nationalité, situation matrimoniale, lieu de résidence, niveau d'instruction et assurance maladie).
- Les circonstances de survenue de la brûlure (sur un lieu de travail ou accident de travail, accidents domestiques, lors de loisirs ou jeux ou pendant les périodes festives, lors de manifestations de rue).
- Les étiologies des brûlures, la nature physique ou chimique de l'agent traumatisant. La brûlure chimique correspondait aux lésions causées par l'action caustique d'un acide ou d'une base forte. Concernant les brûlures physiques, il s'agissait d'un contact de l’œil avec une source électrique ou de chaleur.
- Les données cliniques (meilleure acuité visuelle de loin initiale (MAVLI), signes fonctionnels en particulier douleur, rougeur, baisse d'acuité visuelle, les lésions du globe et des annexes et le stade de la brûlure).
- Les modalités évolutives (meilleure acuité visuelle de loin finale (MAVLF), acuité visuelle de loin finale corrigée, séquelles anatomiques). Elles permettent d'apprécier le pronostic fonctionnel et anatomique.

L'examen ophtalmologique a consisté en la mesure de l'acuité visuelle initiale, suivie de l'examen des annexes du globe oculaire, notamment des culs-de-sac conjonctivaux à la recherche d'un symblépharon. L'examen du segment antérieur permettait la recherche d'une atteinte conjonctivale (hyperhémie conjonctivale, nécrose conjonctivale), cornéenne (érosion/désépithélisation cornéenne, opacité stromale) et limbique (cercle périkératique, blancheur « œil porcelaine »). Ces données physiques permettaient de classer les patients selon la classification de Roper-Hall comprenant 4 stades [12]. Le stade 1 est défini par une désépidermisation cornéenne isolée, un stroma intact et l'absence d'ischémie limbique, et associé à un très bon pronostic. Le stade 2 est défini par une opacité cornéenne, mais les détails de l'iris restent visibles, associés à une ischémie affectant moins du tiers de la circonférence limbique. Le pronostic est bon. Le stade 3 comporte une désépidermisation cornéenne totale, une opacité cornéenne masquant les détails de l'iris et une ischémie affectant entre le tiers et la moitié de la circonférence limbique. Le pronostic est réservé. Le stade 4 est caractérisé par une opacité cornéenne totale sans visibilité des structures du segment antérieur et une ischémie affectant plus de la moitié de la circonférence limbique. Le pronostic est péjoratif.

### Traitement et analyse des données

La saisie et l'analyse des données ont été effectuées dans le respect de l'anonymat des patients et la confidentialité de leurs informations. Un numéro individuel d'identification a été attribué. La saisie et l'analyse des données ont été réalisées avec le logiciel SPSS’ version 23. Les variables quantitatives ont été exprimées sous forme de moyenne, de médiane et de quartile. Les variables qualitatives ont été présentées sous forme de proportion. Les relations entre deux variables ont été évaluées par le test du Khi-deux (χ^2^) avec p < 0,05 comme seuil de signification. Le test exact de Fisher a été utilisé lorsque les conditions d'application de ce dernier n’étaient pas remplies, c'est-à-dire lorsque les effectifs étaient trop petits.

### Considérations éthiques

Cette étude sur dossiers a obtenu l'accord de l’équipe du service d'ophtalmologie du CHU de Cocody. La confidentialité des données a été respectée. Nous avons obtenu l'autorisation des patients pour la prise et la publication des photos dans le cadre scientifique. Cette autorisation des patients a été demandée et accordée au fur et à mesure des soins.

## Résultats

### Aspects socio-démographiques

Durant la période d’étude, 436 patients ont été accueillis dans l'unité de consultation du service d'ophtalmologie du CHU de Cocody pour traumatismes oculaires, dont 49 pour une brûlure oculaire soit une fréquence hospitalière de 11 %. L’âge moyen des patients était de 27,9 ans ± 14,2 avec des extrêmes de 3 et 60 ans. L’âge médian était de 25 ans. Aux premier et deuxième quartiles, les âges étaient respectivement de 18 et 25 ans (Tableau I). Les tranches d’âge majoritaires étaient celles de 0 à 15 ans, 16 à 30 ans et 31 à 45 ans à respectivement 28,6 %, 30,6 % et 30,6 %.

**Tableau I:** Répartition des 49 cas de brûlures oculaires selon les caractéristiques socio-démographiques, CHU de Cocody, 2020-21 Distribution of the 49 cases of eye burns by sociodemographic characteristics, Cocody Hospital, 2020-21

Variables	Effectifs (n)
**Tranche d’âge**	
]0-15 ans]	14
[16-30 ans]	15
[31-45 ans]	15
[46-60 ans]	5
**Total**	**49**
**Sexe**	
masculin	32
féminin	17
**Lieu de résidence**	
hors d'Abidjan	28
Abidjan	21
**Niveau d'instruction**	
analphabète	18
niveau primaire	13
niveau secondaire	11
niveau supérieur	6
niveau préscolaire	1
**Nationalité**	
Ivoirienne	39
Autres nationalités	10
**Assurance maladie**	
non	43
oui	6

Le genre masculin prédominait (32/61 yeux). La majorité des patients résidaient hors d'Abidjan (55 %). Un tiers des patients étaient analphabètes. Dix patients sur 49 étaient non nationaux. La majorité (43 patients) n'avait pas d'assurance maladie. Les élèves et étudiants ainsi que les artisans prédominaient (Tableau II).

**Tableau II:** Répartition des 49 cas de brûlures oculaires selon les activités, CHU de Cocody, 2020-21 Distribution of 49 cases of eye burns according to the activities, Cocody Hospital, 2020-21

Activités des patients	Effectifs (n)
Élèves et étudiants	19
Artisans	8
Commerçants	5
Ménagères	5
Agriculteurs	4
Orpailleurs	4
Autres (sans-emploi, âge préscolaire)	2
Médecin	1
Chef d'entreprise	1
**Total**	**49**

### Aspects diagnostiques

Les brûlures graves (stades 3 et 4) ont été observées chez 36 % des patients (Tableau III). La plupart des brûlures sont survenues lors d'accidents de travail (33 %) ou domestiques (23 %) (Tableau IV).

**Tableau III:** Répartition des yeux selon les stades de brûlures (classification de Roper-Hall), CHU de Cocody, 2020-21 Distribution of eyes by burn stage (Roper-Hall classification), Cocody Hospital, 2020-21

Stade des brûlures	Effectifs (n)
Stade 1	31
Stade 2	8
Stade 3	8
Stade 4	14
**Total**	**61**

**Tableau IV:** Répartition des yeux selon les circonstances des brûlures, CHU de Cocody, 2020-21 Distribution of eyes according to burning circumstances, Cocody Hospital, 2020-21

Circonstances	Effectifs (n)
Jeux	10
Accidents de travail	20
Accidents domestiques	14
Manifestations de rue	7
Travaux champêtres	3
Agressions	3
Soins de beauté	2
Erreurs médicales	2
**Total**	**61**

**Figure 1:**
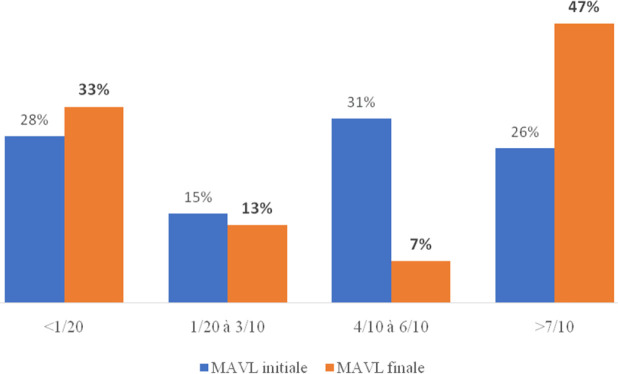
Comparaison entre l'acuité visuelle initiale et finale, CHU de Cocody, 2020-21 Comparison between initial and final visual acuity, Cocody Hospital, 2020-21

Les brûlures chimiques ont été observées chez 54 % de l'effectif et les thermiques dans 46 % des cas (Tableau V).

**Tableau V:** Principales étiologies des brûlures oculaires, CHU de Cocody, 2020-21 Main causes of eye burns, Cocody Hospital, 2020-21

Étiologies	Effectifs (n)
**Brûlure chimique**	
acide pour batterie non dilué	4
eau de Javel	4
soude pour la fabrication de savon (Fig. 2)	5
colle cyanoacrylate	2
eau savonneuse	2
soluté de perfusion sanguine	1
sève d'arbre	5
gaz lacrymogène	7
parfum	2
pommade dermocorticoïde	1
**Total**	**33**
**Brûlure thermique (Fig 3)**	
flamme (Fig. 4)	9
huile chaude	4
feu d'artifice (Fig. 5)	8
aluminium fondu	2
explosif	5
**Total**	**28**

**Figure 2:**
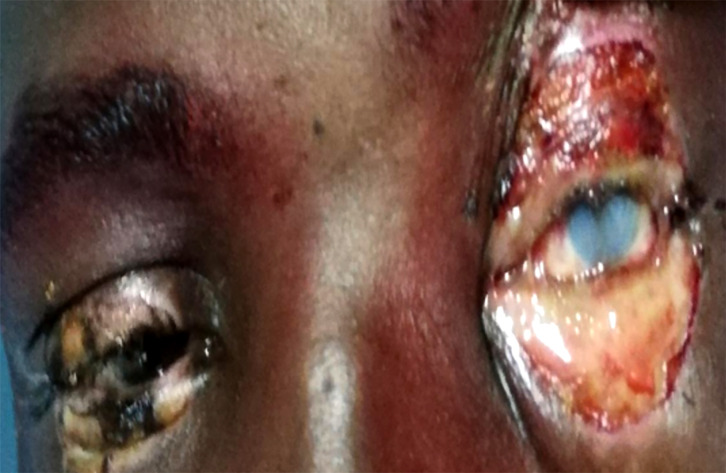
Brûlure oculaire bilatérale par agression avec de l'eau basique pour la fabrication d'un savon traditionnel appelé « kabakrou » Bilateral eye injury from assault with basic water for making a traditional soap called “kabakrou”

**Figure 3:**
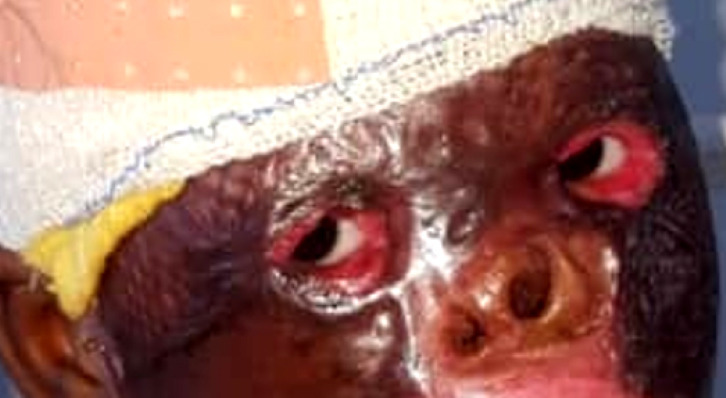
Brûlure thermique prise en charge en collaboration avec le service du grand brulé et en attente de chirurgie palpébrale Thermal burn managed in collaboration with the burn unit and awaiting palpebral surgery

**Figure 4:**
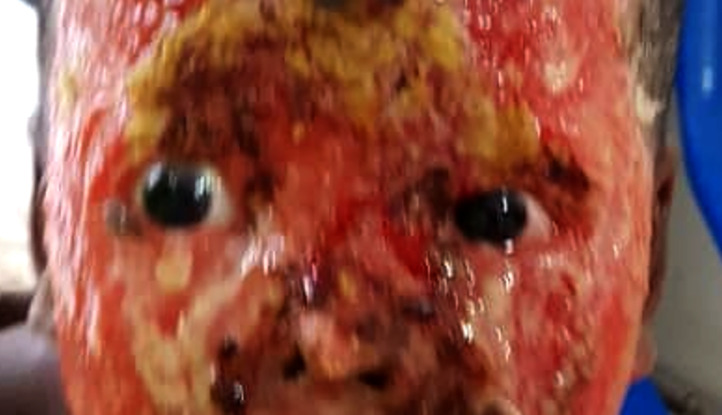
Brûlure thermique par flamme chez un enfant secondaire à une chute dans du feu (atteinte prédominante des cils, sourcils et paupières) Thermal flame burns in a child secondary to a fall into fire (predominantly involving the eyelashes, eyebrows and eyelids)

**Figure 5:**
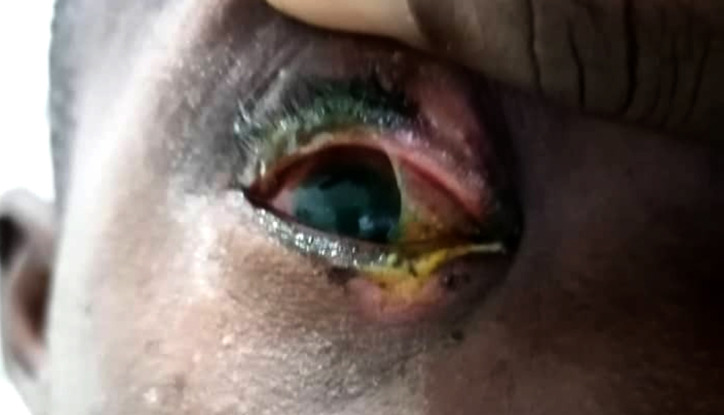
Brûlure oculaire par feu d'artifice avec symblépharon Fireworks eye burn with symblepharon

Le délai moyen de consultation était de 3,5 jours ± 7,9 avec des extrêmes de 1 et 60 jours. Aux premier et deuxième quartiles, le délai de consultation était respectivement de 1 et 2 jours. L'atteinte oculaire était unilatérale droite dans 22 cas (45 %) et gauche dans 15 cas (31 %), bilatérale dans 12 cas (24 %). La MAVLI était inférieure à 1/20 pour 17 yeux (28 %). Des lésions palpébrales (plaies, abrasions, amputation) étaient observées pour 21 yeux (43 %).

### Aspects thérapeutiques

La majorité des cas, soit 34 yeux (55,7 %), a bénéficié de lavage oculaire à l'eau de robinet sur le lieu de l'accident. Tous les patients ont reçu des anti-inflammatoires locaux, des cicatrisants cornéens et des anti-infectieux dans le service d'ophtalmologie du CHU de Cocody. Les cas compliqués d'abcès de cornée ont bénéficié d'anti-infectieux ou d'antibiotiques par voie générale (6 cas). Le traitement chirurgical a concerné 2 cas d’éviscération et 2 cas de chirurgie de symblépharon.

### Aspects évolutifs

L’évolution a été favorable pour 33 yeux (54 %) et défavorable pour 28 : perte définitive de la vision pour 20 yeux (33 %) et malvoyance pour 8 (13 %). La Figure 1 présente la comparaison entre l'acuité visuelle initiale et finale.

Les séquelles étaient des opacités cornéennes dans 21 yeux (34 %) et la phtyse du globe oculaire dans 5 yeux (8 %). Les opacités cornéennes étaient secondaires à des complications infectieuses à type d'abcès de cornée dans 4 cas et d'endophtalmie dans 2 cas. La phtyse était secondaire à un éclatement du globe oculaire (3 cas) et aux complications infectieuses (2 cas). Deux cas de symblépharon et 4 cas de séquelles palpébrales à type de rétraction ont été observés.

### Étude analytique

Les accidents de travail étaient la circonstance de survenue la plus fréquente au cours des brûlures graves (9 cas). Les patients résidant hors d'Abidjan présentaient plus de lésions graves (17 cas). Parmi les patients au stade grave (22 cas), 9 patients (41 %) ont bénéficié d'un traitement sur le lieu de l'accident (lavage à l'eau de robinet) sans lien statistique significatif entre le lavage et le stade de brûlure. Le lavage à l'eau de robinet sur le lieu de l'accident n'est pas statistiquement lié à la gravité des brûlures. Pour un délai moyen de consultation entre 2 et 7 jours, on observait 72 % de stade grave contre 18 % pour un délai moyen de consultation inférieur ou égal à 24 h, sans lien statistique significatif (p = 0,073).

Parmi les cas graves, 89,5 % provenaient de zones rurales (à l'extérieur d'Abidjan). Les brûlures graves étaient observées au cours des accidents par sève d'arbre, feu d'artifice et exposition aux produits de fabrication de savon traditionnel dans des proportions identiques de 18 % avec un lien statistiquement significatif entre les étiologies et le stade des brûlures (p < 10^-3^).

Les stades graves ont évolué vers la cécité chez 86,4 % et la malvoyance chez 13,6 % des patients avec lien statistiquement significatif (p < 10^-3^). Une évolution favorable a été objectivée à 88 % chez les patients ayant consulté dans les 24 premières heures (Tableaux VI et VII).

**Tableau VI:** Relation entre le stade des brûlures, la circonstance de survenue, le lieu de provenance et le délai moyen de prise en charge, CHU de Cocody, 2020-21 Relationship between the stage of burns, the circumstances in which they occurred, the place of origin and the average time of treatment, Cocody Hospital, 2020-21

	Stade de brûlure				Total	Valeur du test	p-value
	Stade 1	Stade 2	Stade 3	Stade 4			
**Circonstances de survenue**							
accidents de travail	8	3	5	4	20	26,7	0,042
travaux champêtres	0	0	0	3	3		
accidents domestiques	10	2	0	2	14		
jeux	3	2	2	3	10		
manifestations de rue	7	0	0	0	7		
soins de beauté	2	0	0	0	2		
erreurs médicales	0	1	0	1	2		
agressions	1	0	1	1	3		
**Total**	31	8	8	14	61		
**Délai moyen de consultation**							
≤ 24 h	17	4	1	3	25	10,2	0,073
2-7 jours	13	3	7	9	32		
> 7 jours	1	1	0	2	4		
**Total**	31	8	8	14	61		
**Lieu de provenance**							
Abidjan	19	3	2	3	27	7,7	0,053
hors Abidjan	12	5	6	11	34		
**Total**	31	8	8	14	61		
**Traitement sur le lieu d'accident**							
oui	19	6	4	5	34	3,9	0,305
non	12	2	4	9	27		
**Total**	31	8	8	14	61		

**Tableau VII:** Relation entre l’évolution, le stade de brûlure oculaire et le délai moyen de consultation, CHU de Cocody, 2020-21 Relationship between evolution, eye burn stage and average consultation time, Cocody Hospital, 2020-21

	Évolution			Total n	Valeur du test	p-value
	Cécité N (%)	Malvoyance N (%)	Évolution favorable avec bonne acuité N (%)			
**Stade de brûlure**						
stade 1	0	1	30	31	71,3	< 0,0001
stade 2	1	4	3	8		
stade 3	5	3	0	8		
stade 4	14	0	0	14		
**Total**	**20**	**8**	**33**	**61**		
**Délai moyen de consultation**						
≤ 24 h	3	2	20	25	12,6	0,006
2-7 jours	15	5	12	32		
> 7 jours	2	1	1	4		
**Total**	20	8	33	61		

## Discussion

Les brûlures oculaires représentent un pourcentage significatif des accidents domestiques (environ 3 à 4 %) et des traumatismes ophtalmiques (7 à 18 %) [5]. Selon notre étude, les brûlures oculaires constituent 11 % de l'ensemble des traumatismes oculaires et surviennent préférentiellement chez les sujets masculins au décours d'un accident du travail. La gravité est corrélée au délai de prise de charge et au stade initial de la pathologie. Les principales limites de cette étude sont inhérentes à sa nature rétrospective : incomplétude des données disponibles (avec manque de données pertinentes), et absence du contrôle des données.

Dans les séries de Domngang [3] et Essid [4], l’âge moyen des patients correspondait à la population active donc celle la plus exposée à son environnement. La prédominance masculine retrouvée dans ce travail avait également été classiquement établie dans la littérature [1, 3, 4]. Cette tendance peut s'expliquer par le fait que les hommes sont plus enclins à exercer des activités exposées aux risques de traumatismes ou adoptent une approche moins prudente que les femmes. La plupart des patients ayant des brûlures graves résidaient hors de la capitale, suggérant un éventuel retard de consultation attribuable à la distance des services d'ophtalmologie. Les zones rurales n'ont en effet pas de service d'ophtalmologie. En conséquence, seuls les patients avec des formes graves vont effectuer le déplacement jusqu’à Abidjan. Concernant les catégories professionnelles, les élèves et étudiants étaient la population prédominante. Au fait d’être jeune et donc plus actif ou relativement imprudent, s'ajoutent les phénomènes de violences en milieu scolaire notamment à l'approche des congés. Les « congés anticipés » consistent à arrêter les cours plus tôt, avant la date des congés scolaires, et perturbent le fonctionnement scolaire. Ce comportement est souvent associé à des incidents violents, mettant en scène des affrontements entre les élèves et les forces de l'ordre ce qui contribue à l'augmentation du risque de traumatisme chez les élèves et étudiants. Les artisans étaient la seconde catégorie professionnelle la plus retrouvée. Ils travaillent fréquemment sans avoir accès à des équipements de protection individuelle (EPI) pour les yeux et le visage, s'exposant ainsi aux traumatismes. Au Cameroun, Koki avait également observé une prédominance d’étudiants et d'artisans dans les traumatismes oculaires [7].

Les accidents du travail représentaient la circonstance de survenue la plus fréquente. Les brûlures chimiques par acides étaient les plus fréquentes. Les acides sont couramment utilisés dans diverses industries, laboratoires, et ménages. Ils sont présents dans de nombreux produits de nettoyage, batteries, et processus industriels, ce qui augmente les risques d'exposition. Ce résultat semble controversé, car les données de la littérature montrent que les lésions par bases sont les plus répandues (dans 2 tiers des cas) [5]. Les acides coagulant les protéines des tissus qui jouent alors un rôle de barrière chimique, la pénétration du produit est stoppée au niveau des couches tissulaires superficielles. Quant aux bases, elles réalisent une saponification des acides gras des membranes cellulaires qui va détruire les cellules de l’épithélium, puis du stroma et de l'endothélium. Elles pénètrent donc plus en profondeur que les brûlures acides. Elles sont parfois redoutables, car malgré un traitement bien conduit, elles peuvent aboutir à la perte fonctionnelle de l’œil. Plus spécifiquement, l'agent traumatisant chimique le plus observé était le gaz lacrymogène (acide). Il s'agit d'un gaz de défense utilisé par les forces de sécurité pour le maintien de l'ordre et le contrôle des foules lors des manifestations de rue et des grèves en milieux universitaire et scolaire. Ce gaz entraîne des irritations oculaires chez les manifestants. Ensuite, une fréquence des lésions par soude caustique et par sève d'arbre était observée. Ces lésions surviennent le plus souvent chez les artisans ou les planteurs qui travaillent sans équipement de protection individuelle.

Les brûlures thermiques représentaient 45,9 % avec, comme agents fréquents, les explosifs (dynamite) et feux d'artifice ou pétards. Les explosions de dynamite surviennent le plus souvent par un déclenchement accidentel en contexte professionnel lors des exploitations minières et des travaux de génie civil. Les feux d'artifice (axés sur des effets visuels spectaculaires) sont souvent utilisés dans des événements plus formels, tandis que les pétards (axés sur la production de bruits forts) sont plus couramment associés à des célébrations informelles. Bien qu'interdit d'utilisation en Côte d'Ivoire y compris en période de fête, hormis dérogation spéciale, ces engins sont communément utilisés lors des célébrations de fin d'année. L’étude d'Ouffoue [10] menée en 2020 montrait la fréquence des traumatismes oculaires par explosifs au cours de cette saison de l'année en Côte d'Ivoire. Toutefois, la sévérité des lésions oculaires par brûlure est également conditionnée par le délai de consultation. Ainsi, dans cette étude, le délai de consultation était relativement tardif pour une urgence. Ce retard de consultation pourrait être imputable à l'influence du contexte culturel africain qui incite à l'utilisation de la tradithérapie en première intention. On pourrait également évoquer l'accès difficile aux centres de santé disposant d'un service d'ophtalmologie et l'insuffisance de moyens financiers de patients ne disposant pas d'assurance maladie. En Côte d'Ivoire, Koffi [6] rapportait, dans 29,4 % des cas, un délai de consultation inférieur à 48 h. En effet, la gravité des brûlures oculaires serait proportionnelle au délai avant la prise en charge initiale, surtout pour les brûlures par bases [9].

Par ailleurs, nos résultats confirment que la consultation précoce (dans les 24 premières heures) est associée à une évolution favorable. Plusieurs autres auteurs ont montré l'importance du lavage oculaire abondant et long (20 à 30 minutes) avec de l'eau sur le lieu de l'accident comme un geste utile dans la prise en charge des brûlures oculaires. Il permet d’éviter la gravité des lésions, surtout pour les brûlures causées par les bases fortes qui sont rapides et irréversibles du fait de leur caractère lipophile [2, 8]. Pourtant, nos résultats n'ont pas montré de lien significatif entre le traitement sur le lieu de l'accident et les stades de brûlure. Ceci pourrait s'expliquer par le fait que le lavage sur le lieu d'accident n'est pas efficace en raison de l’état de panique du patient, du spasme des paupières et de la douleur. Un second lavage oculaire plus efficace sous anesthésie locale a été réalisé lors de la consultation. La majorité des patients avaient reçu uniquement un traitement médical, contre de rares cas ayant bénéficié d'un traitement médico-chirurgical. Le traitement médical visait à mieux contrôler l'inflammation, à favoriser la cicatrisation et à minimiser les risques de complications. Relativement à l’évolution, la cécité monoculaire était retrouvée chez plus d'un quart de l'effectif.

De nombreux cas cliniques ont été rapportés et ont permis de mettre en évidence une mauvaise récupération visuelle chez les patients. Il s'agit de l’étude Rekik [11] qui a relaté un cas de brûlure oculaire avec une acuité visuelle finale à perception lumineuse positive sans récupération visuelle après une kératoplastie transfixiante. Domngang [3] a notifié 3 cas de brûlure dont 2 cas de perception lumineuse positive. Quant à l’étude d'Ouffoue [10] portant sur les brûlures oculaires par pétards et feux d'artifice, elle avait rapporté un pronostic péjoratif dans 75 % des cas avec cécité légale. Dans notre travail, nous avons retrouvé un lien statistiquement significatif entre l’évolution et le stade de brûlure. Ce pronostic fonctionnel pourrait s'expliquer d'une part par le fait que plus d'un tiers des yeux présentaient des lésions de mauvais pronostic et d'autre part, par le délai de consultation long.

De ce qui précède, nous recommandons un renforcement de la loi concernant l'interdiction des engins explosifs, des enseignements sur le civisme dans les établissements scolaires et universitaires, l'organisation de séminaires d'information et de formation sur la santé et la sécurité au travail concernant les EPI. Une sensibilisation média-audiovisuelle doit également être menée à l'endroit des artisans sur l'importance de l'utilisation d'EPI. Améliorer les soins aux patients dans notre contexte pourrait également impliquer l’élargissement de l'accès aux services ophtalmologiques dans chaque région, conjointement avec une sensibilisation à la nécessité des consultations précoces.

## Conclusion

Les brûlures oculaires peuvent être responsables d'une altération profonde et irréversible de la fonction visuelle. Ces lésions sont le plus souvent observées chez les hommes et dans la population active et au décours des accidents de travail. Les stades graves avaient été les moins observés, cependant leur évolution était défavorable. L'un des facteurs de ce mauvais pronostic est le retard de prise en charge des patients.

Afin de prévenir cette affection, l'utilisation de matériel de protection doit être effective dans toutes les professions à risque, notamment les garages, les ateliers, les laboratoires, les entreprises de chimie et de savonnerie. Ensuite, le lavage oculaire occupe également une place prépondérante dans le traitement en urgence et ses règles d'exécution doivent être connues de tous, non seulement des ophtalmologistes, mais aussi des personnels susceptibles de recevoir les urgences oculaires. La prévention à travers l’éducation et l'information des patients est primordiale.

## Contribution des auteurs

KOMAN CE : conception de l’étude, rédaction et validation du protocole, rédaction, relecture et validation du manuscrit

KONAN MANMI SMP : relecture et validation du manuscrit

AGBOHOUN RP : analyse et validation des données, interprétation des résultats

KOUASSI-REBOURS AC, SOWAGNON YTC : relecture et validation du manuscrit

N'DA HC : rédaction du manuscrit

KOUADIO KOUAO CR : prospection bibliographique, rédaction du manuscrit

N'GUESSAN LC : rédaction du manuscrit

KOUASSl FX : supervision de l’étude, relecture et validation du manuscrit

## Liens d'intérêts

Les auteurs ne déclarent aucun lien d'intérêts.
